# Divergent Roles of Autophagy in Virus Infection

**DOI:** 10.3390/cells2010083

**Published:** 2013-01-25

**Authors:** Abhilash I. Chiramel, Nathan R. Brady, Ralf Bartenschlager

**Affiliations:** 1 Department of Infectious Diseases, Molecular Virology, University of Heidelberg, Im Neuenheimer Feld 345, 69120 Heidelberg, Germany; E-Mail: Abhilash_Chiramel@med.uni-heidelberg.de; 2 Systems Biology of Cell Death Mechanisms, German Cancer Research Center (DKFZ), Department of Surgery, Medical Faculty, University of Heidelberg, Bioquant, Im Neuenheimer Feld 267, 69120 Heidelberg, Germany; E-Mail: n.brady@dkfz.de

**Keywords:** selectively autophagy, virophagy, antiviral and proviral autophagy

## Abstract

Viruses have played an important role in human evolution and have evolved diverse strategies to co-exist with their hosts. As obligate intracellular pathogens, viruses exploit and manipulate different host cell processes, including cellular trafficking, metabolism and immunity-related functions, for their own survival. In this article, we review evidence for how autophagy, a highly conserved cellular degradative pathway, serves either as an antiviral defense mechanism or, alternatively, as a pro-viral process during virus infection. Furthermore, we highlight recent reports concerning the role of selective autophagy in virus infection and how viruses manipulate autophagy to evade lysosomal capture and degradation.

## 1. Introduction

To co-exist with their hosts viruses have evolved diverse mechanisms to exploit cellular processes and to evade from host defenses. One such central cell pathway is an ancient degradative process, designated autophagy. In contrast to proteasomal degradation that specifically targets ubiquitinated proteins, autophagy is mediated by the lysosome, which serves as an end point degradative organelle. Autophagy is thus a catabolic process that maintains cellular homeostasis by degradative removal of damaged or excess cellular organelles and protein aggregates from the cytoplasm, thereby enabling cell survival. Both cell culture and *in vivo* studies revealed the fundamental roles of autophagy in numerous diseases, including cancer or neurodegeneration, in aging, but also in innate and adaptive immunity to pathogen infection.

In general, autophagy can be broadly classified as macroautophagy, microautophagy and chaperone-mediated autophagy (CMA). All three pathways share the same mode of degradation via the lysosome, but are mechanistically distinct from each other [1]. Microautophagy, mainly characterized in yeast [2], involves a direct engulfment of the cytoplasm at the lysosomal membrane, facilitated by protrusion of arm-like structures, and CMA specifically directs translocation of unfolded proteins with an inherent consensus motif (KFERQ) across the limiting membrane of the lysosome for degradation [1]. Furthermore, a crucial component for CMA is the lysosomal membrane receptor, lysosome-associated membrane protein (LAMP) type 2A [1]. In contrast to CMA, macroautophagy (called autophagy henceforth) involves sequestration of intact organelles (e.g., mitochondria) and portions of the cytosol via a membrane referred to as the phagophore or isolation membrane [3,4]. This phagophore expands to form double-membrane vesicles, termed autophagosomes [3,4]. Subsequently, autophagosomes mature by fusing with endosomes and/or lysosomes to form autolysosomes, where degradation of the internal contents occurs by resident lysosomal hydrolases [5]. The membrane source for the biogenesis of the phagophore is debatable; however, several recent studies have shown that the endoplasmic reticulum (ER), mitochondria and plasma membrane (PM) serve as source for the phagophore [6,7,8].

**Figure 1:**
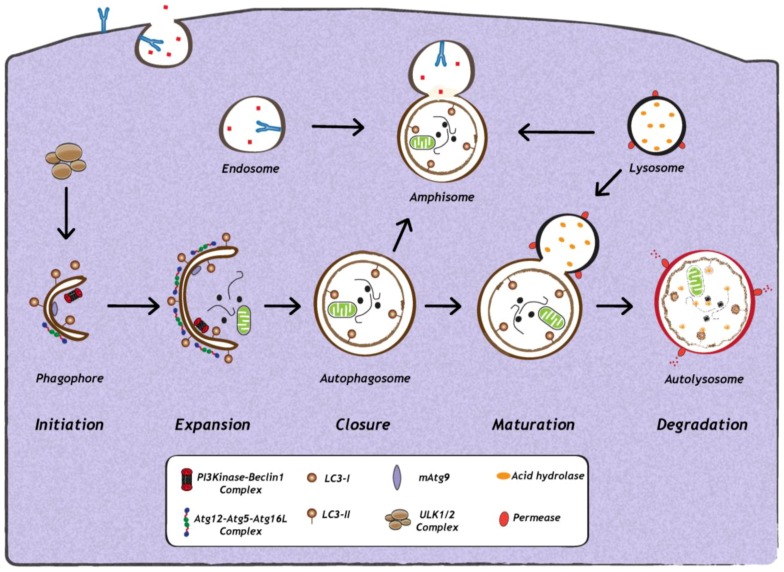
Schematic representation of autophagy. The first step in the initiation of mammalian autophagy is the formation of the phagophore, followed by subsequent steps, which include elongation and expansion of the phagophore, closure and completion of a double-membrane autophagosome (which engulfs a portion of the cytoplasm), autophagosome maturation via docking and fusion with endosomal compartments (to form a hybrid vesicular product known as an amphisome) and/or with a lysosome (to form the autolysosome). The inner autophagosomal membrane and cargo is degraded by acid hydrolases inside the autolysosome, and recycling of the resulting macromolecules is mediated via permeases. The molecular machinery regulating autophagy is also depicted. ULK1 and ULK2 complexes that are crucial for induction of autophagy, PI3Kinase-Beclin1 complexes, are required for autophagosome formation, the delivery of membranes to the forming autophagosome is mediated by mammalian Atg9 (mAtg9) and two conjugation systems: LC3-II and Atg12–Atg5–Atg16L complex, which are required during elongation and expansion of the phagophore.

Although the autophagy concept was developed more than 50 years ago [1], insights into the molecular mechanisms came to light only in the late 1990s from yeast genetic screens that identified mutants of autophagy-related genes (Atgs). These yeast-specific Atgs provided great insight into the molecular aspects of autophagy in higher eukaryotes. Moreover, the first identified mammalian Atgs, Atg5 and Atg12, were shown to be highly homologous to those in yeast [1]. Furthermore, the morphology of autophagosomes in yeast is similar to those in mammals [9]. The majority of proteins involved in the autophagy machinery are required for formation and elongation of the phagophore [1] (Figure 1). Moreover, the entire pathway of autophagy, from the formation of autophagosomes to their fusion with endosomes and lysosomes, is highly dynamic.

Autophagy can operate either as a non-selective or a highly selective process: first, a starvation induced, non-selective pathway; second, a target-specific and selective autophagic pathway [10]. Non-selective or starvation-induced autophagy is thought to play an important role in supplying energy to the cell by the bulk degradation of proteins and organelles, whereas selective autophagy involves the recruitment of specific adaptor proteins such as p62, NBR1 (neighbor of BRCA1 gene 1), BNIP3 (BCL2 and adenovirus E1B 19 kDa-interacting protein 3) and NIX (NIP3-like protein X) that recognize ubiquitinated protein complexes and organelles, such as mitochondria (mitophagy), peroxisomes (pexophagy) and ribosomes (ribophagy), to target them to autophagosomes for degradation [10,11,12]. More recently, studies have shown that selective autophagy plays a crucial role in the elimination of pathogenic bacteria and viruses, termed xenophagy, thus extending the functions of autophagy to innate and adaptive immunity against pathogens [11]. Moreover, studies on selective autophagy have underlined the relevance of post-translational modifications, such as phosphorylation and ubiquitination, in linking autophagy adaptor protein function to autophagy substrate recognition [13,14]. However, the role of adaptor proteins in non-selective autophagy still remains to be fully determined.

In this review we will discuss the current understanding of how virus infection regulates the crosstalk between intracellular signaling and autophagy. Furthermore, we will highlight recent reports on how autophagy exerts its antiviral function and the countermeasures adopted by viruses to exploit and subvert autophagy.

## 2. Impact of Viral Infection on Autophagy

Autophagy induction involves the coordinated action of several key factors (Figure 1). Among them are members of the Atg8 ubiquitin-like (Ubl) protein family, which include MAP1LC3A (microtubule-associated protein 1 light chain 3A), MAP1LC3B, MAP1LC3C, GABARAP (у-aminobutyric acid type A [GABA] receptor-associated protein), GABARAPL1 (GABARAP—like 1), GABARAPL2 and GABARAPL3, all of which are important for phagophore formation and closure in mammals [15]. In principle, any of the above mentioned Atg8 family proteins can serve as a marker of autophagosomes in mammalian cells, however, conventionally only MAP1LC3B (referred to as LC3 henceforth) is used as marker of autophagosomes in mammalian cells [16,17]. During autophagosome formation and elongation, the cytosolic LC3, termed LC3-I, is proteolytically cleaved and coupled to phosphatidyl-ethanolamine (PE) to form LC3-II, which is inserted into the autophagosomal membrane [1,9]. The two main characteristics of autophagy detection is, first, the increase in intracellular levels of LC3-II and, second, the re-localization of evenly distributed cytosolic LC3-I into LC3-II positive punctuate structures, representing autophagosomes [1,9]. Indeed, infections with a wide range of DNA or RNA viruses increase abundance of autophagosomes or autophagic vesicles in infected cells [18,19,20,21,22,23,24,25,26,27,28,29,30,31,32,33,34]. Furthermore, several electron microscopy-based studies revealed the presence of double-membrane structures that are characteristic of autophagosomes. However, it is important to note that increased amounts of autophagosomes in infected cells can either be due to their enhanced formation or to their accumulation due to a block in their maturation or degradation. Therefore, to understand the reason for increased autophagosome numbers in virus-infected cells, it is important to study the impact of viral infection on autophagic activity or autophagic flux, which is defined as the measurement of the balance between the rate of autophagosome formation and degradation. Assays to measure autophagic activity in mammalian cells have been described in detail in other reviews and, therefore, will only be mentioned here [16,17]. 

### 2.1. Induction and Inhibition of Autophagy by Viruses

Various studies reported the activation of autophagy upon virus infection as inferred from the increased number of autophagic vesicles, the monitoring of LC3-I to LC3-II conversion and the elevated number of LC3-positive punctae in infected cells [21,22,23,29,30,32,33,35]. However, considering the dynamic nature of autophagy, it is important to determine the rate of autophagic flux to gain mechanistic insight into how viral infection affects autophagic activity in cells. In fact, recent reports demonstrated that Sindbis virus and hepatitis C virus (HCV) infection increase autophagic flux [36,37]. Infection of mouse embryonic fibroblasts (MEFs) expressing GFP-LC3 with Sindbis virus resulted in an increase in the percentage of cells containing GFP-LC3 positive dots concomitant with an increase in LC3-II abundance. In addition, infected cells contained reduced levels of p62 (an accepted marker of autophagic flux), with no significant changes of its mRNA amounts, indicating a complete autophagic response during virus infection [37]. In case of HCV infection, a study by Ke and Chen [36] demonstrated that despite unaltered levels of p62 in infected cells, LC3-II protein levels were increased upon treatment with lysosomal protease inhibitors (E64 and pepstatin A), or chloroquine (a vacuolar ATPase inhibitor) or bafilomycin A1 (an inhibitor of lysosomal acidification), [36] arguing that HCV does not block autophagy. In line with this conclusion, the authors showed that treatment of infected cells with chloroquine and bafilomycin A1 resulted in increased LC3-II levels in HCV infected cells as compared to uninfected cells, clearly indicating enhanced autophagic flux. In addition, by employing a tandem-LC3 reporter (mRFP-GFP-LC3), a tool to analyze both autophagosome and autolysosome formation, the authors found that HCV induced autophagosomes that matured into autolysosomes, thus revealing a complete autophagic response in HCV-infected cells. In contrast, an earlier study by Sir and co-workers proposed that autophagy is incomplete, and cells transfected with HCV RNA formed autophagosomes that, due to inefficient fusion with lysosomes, did not convert into autolysosomes [23]. The discrepancy between these two studies might be due to differences in the experimental approaches: HCV infection *versus* electroporation of HCV RNA.

**Figure 2:**
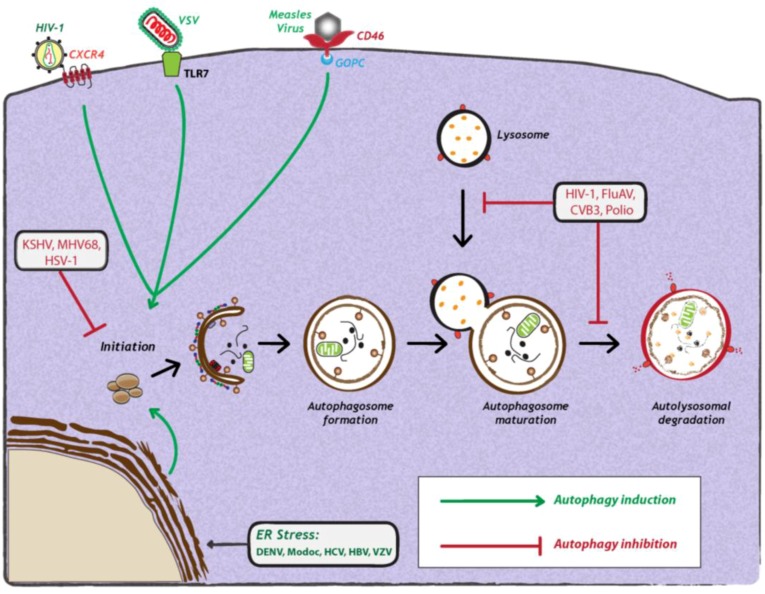
Viral manipulation of autophagy. Viruses can either induce (green arrows) or inhibit the autophagy pathway (red studded lines). Viral induction of autophagy can be achieved during viral entry via interaction with cell surface receptors, via interaction with stress sensors or during viral replication. Viruses also inhibit autophagy at early or late stages of the pathway, during initiation or maturation, respectively. For further details, see the legend for Figure 1. Abbreviations: HIV-1 (human immunodeficiency virus-1); CXCR4 (C-X-C chemokine receptor type 4); VSV (vesicular stomatitis virus); TLR7 (Toll-like receptor 7); CD46 (cluster of differentiation 46); GOPC (Golgi-associated PDZ and coiled-coil motif-containing protein); KSHV (Kaposi’s sarcoma herpesvirus); MHV68 (murine γ-herpesvirus 68); HSV-1 (herpes simplex virus-1); FluAV (influenza A virus); CVB3 (coxsackievirus B3); DENV (dengue virus); HCV (Hepatitis C virus); HBV (Hepatitis B virus); VZV (varicella-zoster virus) ER (endoplasmic reticulum).

As opposed to Sindbis virus and HCV, a number of viruses have evolved strategies to interfere with autophagosome formation or maturation (Figure 2). For instance, Kaposi’s Sarcoma herpesvirus (KSHV), murine γ-herpesvirus 68 (MHV68) and Herpes Simplex virus 1 (HSV-1) target Beclin-1, thereby inhibiting autophagosome formation. These viruses encode proteins that competitively bind to Beclin-1 and thus inhibit its interaction with Vps34. KSHV encodes a viral Bcl-2 homologue, which binds Beclin-1 with much higher affinity than cellular Bcl-2, thereby preventing incorporation of Beclin-1 into Vsp34 complexes [38]. M11, the viral Bcl-2 homologue encoded by MHV68, also interacts with Beclin-1 and inhibits autophagosome formation [39]. In the case of HSV-1, the virally encoded protein ICP34.5 interacts with Beclin-1 via a 20 amino acid residues-long region (aa 68-87) to inhibit autophagosome biogenesis [40]. Interestingly, deletion of ICP34.5 leads to accumulation of HSV-1 particles in autophagosome-like structures, which can be visualized by electron microscopy [41]. Other viruse,s such as human immunodeficiency virus-1 (HIV-1), influenza A virus (FluAv), coxsackievirus B3 (CVB3) and poliovirus inhibit autophagy by blocking autophagosome maturation or degradation. In the case of HIV-1 and FluAv, the viral proteins Nef and M2, respectively, block autophagosome maturation by a still poorly defined mechanism that depends on their interaction with Beclin-1 [27,28]. Studies with CVB3 and poliovirus show that autophagy is inhibited at the stage of autolysosomal degradation [18]. Moreover, in the case of coxsackievirus B3 (CVB3) [25,42], it was demonstrated that protein levels of LC3-II and p62 increase over time, suggesting that viral infection leads to an accumulation of autophagosomes due to a block in autolysosomal degradation [25,42]. However, it remains to be determined how these viruses specifically target autolysosomal degradation.

### 2.2. Cellular Signaling Events Influencing Autophagy During Viral Infection

The induction of autophagy is triggered by a variety of stress stimuli, including nutrient deprivation, ER stress, danger-associated molecular patterns (DAMPs), hypoxia, redox stress and mitochondrial damage. These stimuli involve a diverse range of cellular signals that have overlapping functions in autophagy and the control of other cellular stress responses. Viruses can trigger many of these stimuli during different stages of their replication cycle. For instance, several reports have shown that upon binding to the surface of target cells, some viruses can stimulate autophagy. One example is CD46 (cluster of differentiation 46), a cell surface receptor which is recognized by several viral pathogens, including human herpesvirus 6, adenovirus types B and D, and measles virus [43] (Figure 2). In case of the latter, CD46 engagement was shown to be sufficient to induce autophagy; viral binding of CD46 leads to its interaction with GOPC (Golgi-associated PDZ and coiled-coil motif-containing protein) (Figure 2), which then associates with the Vps34-Beclin-1 complex, leading to activation of autophagy [43]. While it is not known whether other viruses entering cells via CD46 also induce autophagy, in the case of HIV-1 binding to CD4+, T-cells trigger autophagy, and this is mediated by the C-terminal domain of the fusogenic gp41 subunit of the viral envelope protein [44,45]. Recent studies with the vesicular stomatitis virus (VSV) demonstrated that virus binding to Drosophila S2 cells was required to induce autophagy [31], which occurred via down regulation of mTOR activity and inactivation of Akt signaling [31]. Finally, a very recent study demonstrated that VSV-induced autophagy depends on Toll-7 in S2 cells and in adult flies [46]. The authors show that VSV interacted with Toll-7 at the plasma membrane to induce antiviral autophagy independent of the canonical Toll signaling pathway [46] (Figure 2).

Apart from attachment of the virus particle to the host cell, viral replication itself frequently elicits stress responses, such as ER stress or production of reactive oxygen species (ROS) that induce autophagy [47]. The endoplasmic reticulum (ER) is the major site for protein synthesis. An accumulation of misfolded or unfolded proteins in this compartment results in ER stress and activates an unfolded protein response (UPR) mediated by three ER membrane-associated proteins: PERK (PKR-like eIF2α kinase), ATF6 (activating transcription factor-6) and IRE1 (inositol requiring enzyme 1). All three proteins are normally bound to and inactivated by the chaperone Bip/GRP78 at the side of the ER lumen. When ER stress is triggered, Bip interacts with unfolded luminal proteins, thus leading to the release of PERK, IRE1 and ATF6. PERK mediates the phosphorylation of eukaryotic translation initiation factor 2 α (eIF2α), resulting in rapid reduction of mRNA translation, thereby reducing the load of new proteins in the ER [48]. In addition, PERK phosphorylation of eIF2α allows the translation of activating transcription factor 4 (ATF4) to induce transcription of genes involved in amino acid synthesis and apoptosis [48]. IRE1 is autophosphorylated to function as an endoribonuclease, responsible for the unconventional splicing of the X box-binding protein 1 (XBP1) mRNA. The resulting translation product XBP1 regulates transcription of genes encoding for ER chaperones, biogenesis of phospholipids and components of the ER-associated protein degradation (ERAD) machinery. Finally, activated ATF6 translocates from the ER to the Golgi to undergo cleavage by regulated intramembrane proteolysis (RIP) by site 1 and site 2 proteases. The cytoplasmic domain of ATF6 functions as a transcription factor to transactivate genes encoding ER chaperones, ERAD components and protein foldases [48]. 

Among these, PERK and ATF6 function as inducers of autophagy, while IRE1 acts as a negative regulator [47]. It was recently shown that HCV infection can induce ER stress and activate all three sensors of UPR [47]. siRNA-mediated silencing of any of the three UPR signaling proteins not only resulted in a significant reduction of LC3-II abundance, but also inhibited HCV replication, arguing that all three UPR signaling pathways are required for HCV-induced autophagy [36] (Figure 2). The exact mechanism by which HCV mediates induction of autophagy via ER stress and UPR remains to be investigated. In contrast, a very recent study by Mohl *et al* provided evidence that HCV-induced autophagy is independent of UPR [49]. Subgenomic replicons expressing nonstructural (NS3-5B) proteins, as well as a mutant virus lacking the envelope glycoproteins, potently induced autophagy in the absence of detectable UPR [49]. Thus, taking both studies together [36,49], it is unclear how autophagy is induced in HCV infection. Recent studies with other related flaviviruses, such as Dengue virus (DENV) and Modoc virus, showed that virus infection induced autophagy [50]. In fact, expression of the replicase protein NS4A from either virus was sufficient for autophagy induction via UPR [50] (Figure 2). However, the impact of these viral proteins on autophagic flux remains to be determined. Lastly, DNA viruses can also induce autophagy via ER-stress and UPR-related pathways. In the case of varicella-zoster virus (VZV) infection, it was demonstrated that virus-induced ER stress and UPR response precede autophagosome formation [51]. The authors show that ER stress upon VZV infection is due to abundant VZV glycoprotein biosynthesis, which in turn triggers to UPR and thus, autophagy to maintain cellular homeostasis.

## 3. Relationship Between Autophagy and Viruses

Depending on the virus and the host cell, autophagy-mediated responses can have different effects on the outcome of viral infections. As an integral part of the immune system, autophagy is involved in the sensing of viruses and mounting of an antiviral defense. It is therefore of little surprise that viruses have evolved mechanisms to subvert the autophagic response.

### 3.1. Antiviral Functions of Autophagy

**Figure 3:**
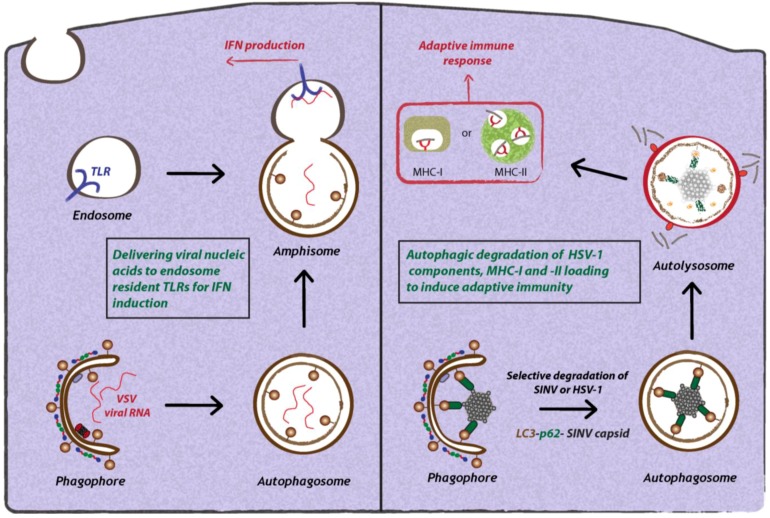
Antiviral functions of autophagy during virus infection. Autophagy can restrict viral replication via different mechanisms. Autophagy selectively transfers viral nucleic acids to endosomal compartments to stimulate innate immune responses via TLRs. Furthermore, autophagy can selectively target viral components and degrade them in lysosomes. Lastly, autophagy mediates the delivery of viral antigens to MHC-I and -II complexes to allow antigen presentation.

As an organelle gathering cytosolic content into a double-membrane vesicle that fuses with endosomal compartments, the autophagosome can specifically deliver intracellular pathogen-associated molecular patterns (PAMPs) to endosomal pattern recognition receptors (PRRs) and MHC-loading compartments to initiate innate and adaptive immune responses (Figure 3). Moreover, the autophagosome can selectively target virions and virus components for degradation via the autolysosome. Both aspects will be discussed in the following paragraphs.

#### 3.1.1. Interplay Between Toll-like Receptors (TLRs) and Autophagy During Viral Infection

The innate immune system is considered as the first line of defense against invading pathogens like viruses and bacteria. Upon viral infection, many cytosolic and endosomal immune sensors detect different components of viral nucleic acids to elicit an innate immune response. There are three classes of PRRs or immune sensors: Toll-like receptors (TLRs), retinoic acid-inducible gene I (RIG-I)-like receptors (RLRs) and nucleotide oligomerization domain (NOD)-like receptors (NLRs) [52]. TLRs are located either on the plasma membrane or within endosomal compartments. In contrast, RLRs and NLRs are mainly localized in the cytoplasm. Among the TLRs, TLR3, 7/8 and 9, which are mainly localized in endosomal compartments, recognize dsRNA, ssRNA and CpG DNA, respectively [52]. Many RNA viruses release their viral RNA genome into the cytoplasm to establish sites of RNA replication, thus allowing cytosolic PRRs to be activated [53]. However, activation of endosomal TLRs can be limited, because viral RNA is packed into the nucleocapsid or sequestered in membrane-associated replication complexes and thus, well protected from the endosomal environment. Nonetheless, TLRs frequently trigger an immune response targeting a number of viral infections via poorly defined mechanisms [54]. 

The first report demonstrating that autophagy can be involved in delivering PAMPs or viral nucleic acids to endosomal TLRs comes from the study of VSV-infected plasmacytoid dendritic cells (pDCs) [55] (Figure 3). The authors showed that the production of interferon-α (IFN-α) by VSV-infected pDCs depended on the autophagic delivery of viral replication intermediates to TLR7 present on late endosomes. This activation of IFN-α production required active viral replication. Furthermore, upon treatment of VSV-infected pDCs with Wortmannin or 3-methyladenine (3-MA) that both inhibit Vps34 activity, IFN-α production was blocked. Finally, the authors showed that Atg5-/- mice exhibited a similar phenotype and carried higher viral loads, suggesting that autophagy is important in controlling VSV replication *in vivo*. However, the exact mechanism by which viral PAMPs are selectively targeted by autophagosomes to the TLR-resident late endosomal compartment remains to be determined.

The involvement of autophagy in delivering viral PAMPs to endosomal PRRs is also conserved in non-immune cells. Studies with Coxsackie virus B3 (CVB3)-infected kidney fibroblasts demonstrated autophagy-dependent activation of TLR3 [56]. The same study revealed that TLR3-decorated vesicles colocalized with LC3-positive structures and that the activation of TLR3 via poly (I:C) or CVB3 infection was sensitive to inhibitors of autophagy induction, autolysosomal acidification and lysosomal proteases. Furthermore, even in the absence of poly (I:C) or CVB3 infection, TLR3 still localized to LC3-positive structures, arguing that even under basal conditions, the autophagosome might fuse with TLR3-positive endosomal vesicles [56]. The resulting ‘mixed’ compartments might therefore serve as antiviral platforms specialized in sensing viral PAMPs and eventually triggering downstream signaling events.

Induction of autophagy can also occur via direct activation of TLRs [57]. Upon stimulation of TLR 3, 4 and 7 with different purified PAMPs, autophagy was shown to be induced in a MyD88- and/or TRIF-dependent manner [58,59]. MyD88 and TRIF function as adaptors through which TLR signaling is mediated. Upon TLR activation, MyD88 and TRIF directly interact with Beclin-1 leading to the formation of Beclin-1-Vsp34 complexes that induce autophagy [59].

The role of autophagy in delivering viral PAMPs to TLRs and the subsequent induction of autophagy upon TLR activation indicates the existence of a positive feedback loop. This feedback loop might not only play an important role in the autophagic sequestration and delivery of PAMPs to endosomal compartments, but also serve for autolysosomal degradation of virions and viral proteins, as well as for antigen presentation via MHC-I and MHC-II molecules, thereby triggering the adaptive immune response.

#### 3.1.2. Autophagic Degradation of Viruses by SLRs (Sequestosome 1/p62-like Receptors)

Autophagy adaptors, referred to as SLRs, are considered as PRRs that can selectively target a variety of pathogens for autophagic degradation [60]. The cytoplasm offers a diverse range of autophagic targets that vary in size and complexity, ranging from protein aggregates up to complete organelles, which can be selectively recognized and sequestered by proteins that function as autophagic adaptors [60]. The main autophagic adaptors that are classified as SLRs in response to bacterial and viral infections, include p62, NBR1 (neighbor of BRCA1 gene 1), NDP52 (nuclear dot protein 52 kDa) and optineurin [60]. Typically, SLRs contain cargo recognition and capture domains, LC3-interacting regions (LIR) to target captured cargo (bacteria or viruses) to the autophagosomal compartment and additional protein interaction domains that are involved in inflammatory processes [60]. However, in the case of Salmonella infection, it was demonstrated that pathogen recognition and capture occurs via multiple SLRs, such as p62, NDP52 and optineurin, by recognition of conventional or branched ubiquitin chains that were associated with or in close proximity of cytosolic salmonellae [60,61]. 

The role of SLRs in the selective autophagic degradation of viruses was recently studied in the context of Sindbis virus infection [37]. Orvedahl and co-workers found that autophagic degradation of Sindbis virus involves the selective degradation of the viral capsid in a p62-dependent manner (Figure 3). The precise signal for capsid recognition by p62 is unclear, but is independent of ubiquitin association, because neurons of infected wild-type or Atg5-/- mice did not contain accumulations of ubiquitinated cellular or viral proteins [37]. Moreover, in an effort to identify host cell factors that target Sindbis virus and HSV-1 for autophagic degradation, the authors performed a genome-wide RNA interference screen. Surprisingly, this screen identified common molecular determinants involved in the targeting of viral nucleocapsids and mitochondria for autophagic degradation [62]. This virophagy and mitophagy is critically determined by SMURF1, an E3 ubiquitin ligase that acts as a bona fide mediator of selective autophagy [62]. SMURF1 might therefore be considered as a novel SLR specifically involved in controlling viral infections. Whether SMURF1 is also involved in autophagy of bacterial invaders remains to be determined.

#### 3.1.3. Role of Autophagy in Antigen Presentation

The role of autophagy is not limited to the innate immune response, but also plays a major role in the adaptive immune response by processing and delivering antigens for presentation in complex with MHC-I and MHC-II molecules [63] (Figure 3). Typically, MHC-I molecules present endogenous antigens that are degraded by the proteasome and transported into the ER, where they are loaded onto MHC-I molecules. After transport to the cell surface, peptide-loaded MHC-I stimulates a CD8+ T-cell response responsible for controlling infections [64]. MHC-II molecules present exogenous antigenic peptides that are processed by lysosomal degradation. Peptides are loaded onto MHC-II and transported to the cell surface to stimulate a CD4+ T- cell response [64]. Notably, exogenous antigens can also be presented on MHC-I molecules to mediate a CD8+ T-cell response by a mechanism termed “cross-presentation” [65]. This is found primarily with dendritic cells, macrophages and B lymphocytes and plays a major role in the immune defense against numerous viral and bacterial infections.

A number of studies have convincingly demonstrated that autophagy can influence the presentation of viral antigens on MHC-I and MHC-II [66]. Studies with HSV-1 showed that at early time points after infection, autophagy is not required for viral antigen processing and presentation of MHC-I peptides, but comes into play at late stage of infection [67]. However, it remains unknown how degradation of HSV-1 particles by the autophagosome results in the presentation of viral antigens to ER localized MHC-I molecules. In the case of Epstein Barr virus (EBV), the viral protein EBNA1 is targeted to MHC-II compartments via autophagic uptake and fusion of autophagosomes with these compartments [68]. Inhibition of autolysosomal acidification resulted in enrichment of EBNA1-positive autophagosomes, and knock-down of Atg12 rendered the cells unable to stimulate MHC-II-dependent activation of T-cells [69]. Importantly, already under normal conditions and in the absence of viral infection, autophagy was required for the consecutive delivery of antigens to MHC-II molecules, arguing for a crucial role of autophagy in MHC-II presentation of antigens *in vivo* [70]. Furthermore, by fusing the matrix protein of influenza virus to LC3, an increased MHC-II activation of CD4+ T-cells was observed, thus revealing an unexplored strategy for vaccine development [70].

### 3.2. Proviral Functions of Autophagy

Apart from playing a major role in antiviral defense, it is becoming increasingly clear that autophagy or individual factors of the autophagy pathway can also enhance viral replication. Many viruses have evolved strategies to directly or indirectly subvert autophagy in order to promote different stages of the viral life cycle. In fact, studies involving pharmacological inhibition or gene silencing of the autophagy pathway demonstrated inhibition of replication and spread of viruses, whereas viral induction of autophagy results in enhanced virus yields.

#### 3.2.1. Role of Autophagy in Promoting Viral Replication

**Figure 4:**
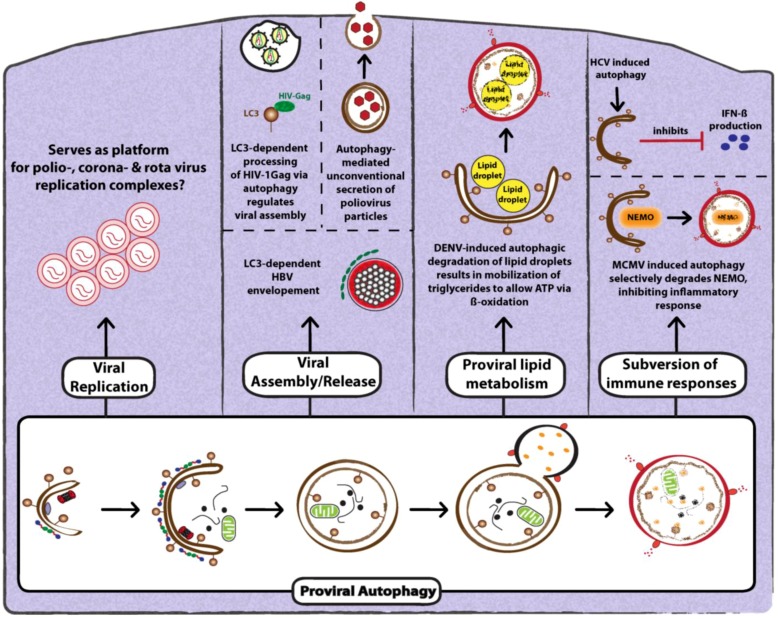
Proviral functions of autophagy. Autophagy is known to exert diverse proviral functions during viral infection. Autophagosomes have been proposed provide a membrane platform for viral replication complexes or to mediate virus assembly and release. Furthermore, viruses can trigger selective autophagy to degrade either lipids (lipid droplets) for energy production during viral replication or to subvert immune responses by selectively degrading key regulatory molecules. The mechanistic details related to proviral functions of autophagy are discussed in the text.

Most positive-strand RNA viruses re-shape the endomembrane system in order to create membrane-associated replication ‘factories’ [71]. This reorganization of intracellular membranes frequently requires autophagy and was first described for the poliovirus [72]. By using thin section EM analysis of infected cells, double-membrane vesicles (DMVs) were detected that are characteristic of autophagosome-like structures (Figure 4). These structures stained positive for the viral protein 2BC, and they did not co-fractionate with markers of other known organelles, highlighting their distinct nature [72]. Expression of the viral proteins 2BC and 3A resulted in the lipidation of LC3 and induced the formation of autophagosome-like DMVs [72]. These properties are not unique for poliovirus, but have been found for several other picornaviruses such as CVB3, enterovirus 71 (EV71) and foot-and-mouth disease virus (FMDV) [42,73,74]. Cells infected with these viruses also contain accumulations of DMVs that are not only morphologically similar, but also contain LC3 as well as viral replicase proteins (Figure 4). However, it is important to note that the membrane composition of picornaviral replication sites is heterogeneous, and other mechanisms have been suggested to explain their formation and composition [75,76]. Importantly, key elements of the secretory pathway are involved in the biogenesis of these membranous structures, including COP-II coated vesicles, Golgi-specific brefeldin A-resistant guanine nucleotide exchange factor-1 (GBF-1), ADP-ribosylation factor 1 (ARF1) and phosphatidylinositol 4-kinase III beta (PI4KIIIβ) [77]. Finally, it was also shown that poliovirus, EV71 and FMDV can replicate in the absence of autophagy, although in some cases virus production was decreased [18,42,73,78]. These studies suggest that autophagosomes, per se, are dispensable for the biogenesis of viral replication sites, although some autophagy proteins can contribute to their morphological heterogeneity.

Conflicting reports have been published as to the role of autophagy for the HCV replication cycle [23,26,79,80]. For instance, Sir and co-workers used the highly permissive cell clone Huh 7.5 and found that transfection of HCV RNA induces autophagic vacuoles and leads to an incomplete autophagic process that promotes viral replication [23]. Dreux and co-workers employed Huh7 cells infected with HCV and found that autophagy was only required for initiating the translation of incoming viral RNA, but not for establishment or maintenance of viral replication [26]. Moreover, in Huh7 cells containing HCV replicons of different genotypes (1a, 1b or 2a), the knock down of Atg proteins did not affect expression of viral proteins [81]. In contrast, in another study that employed Huh7.5 cells, the knock-down of Atg7 dramatically decreased production of HCV particles, but did not affect viral replication [80]. Moreover, a recent report showed that knock-down of the autophagy-related proteins beclin 1 (BCN1) and Atg7 inhibited HCV replication in immortalized human hepatocytes (IHHs) [82]. These conflicting results might be due to the use of different cell lines. In fact, a recent study highlighted the importance of the UPR and autophagy during HCV infection and demonstrated that autophagy enhances HCV replication by inhibiting an innate immune response [36]. Since Huh7 clones differ in their capability to mount an innate antiviral defense, the use of different cell lines could explain, at least in part, the contradictory results.

More recently, Sir et al proposed a direct role of autophagy in serving as platforms for viral RNA replication [79]. In this study, the authors describe the localization of HCV replicase proteins NS5A and NS5B as well as nascent HCV RNA to autophagosome-like structures. However, these data are in direct opposition to studies showing that HCV proteins do not colocalize with autophagosomes in infected cells [23,49,80]. Thus, although autophagy is required for HCV replication, the precise role of autophagy or autophagy-related proteins in viral replication remains to be determined.

In the case of coronaviruses, autophagosomes were initially proposed to be sites of RNA replication, since viral replicase proteins colocalized with LC3 and the deletion of Atg5 restricted viral replication [83]. However, subsequent studies suggested that the role of autophagy in viral replication might be a cell type-specific effect [84]. Electron tomography and 3D reconstructions of coronavirus-infected cells demonstrated that DMVs form an adjoining network likely derived from the ER [85]. Moreover, LC3 was localized to the viral replicase machinery, and surprisingly, only unlipidated LC3-I, but not LC3-II, decorated the replication machinery, as well as replication membranes [86]. Furthermore, viral DMVs also stained positive for ER-associated degradation (ERAD) markers. This data suggests that coronaviruses do not require autophagosomes, per se, but utilize components of the autophagy pathway. Another example of viruses utilizing autophagy for viral replication is rotavirus. It was found that the viral protein NS4P, a marker of rotavirus replication sites, colocalized with LC3-positive structures [19] (Figure 4). These structures did not stain positive for lamp1, suggesting a block in fusion of virus-associated autophagic structures with lysosomal compartments [19]. However, the precise role of autophagy in viral replication remains to be explored. 

Apart from a direct contribution to the formation of membranous replication factories, autophagy can promote viral replication in other ways. For instance, a recent study by Heaton and co-workers identified a novel proviral mechanism of autophagy: the production of energy [87]. Taking into account the so-called lipophagy, *i.e.,* the degradation of triglycerides stored in lipid droplets (LDs), the authors demonstrated that DENV infection of hepatocytes induces autophagy-mediated LD degradation. This results in a mobilization of triglycerides that are used for ATP production via β-oxidation. The signaling pathway controlling the selective degradation of LDs via autophagy and the mechanism that regulates ATP production are not clear. Nevertheless, this study is the first to link of viral infection to autophagy-dependent regulation of lipid metabolism. It will be interesting to determine whether this link is unique to DENV or applies to other (LD-dependent) viruses.

#### 3.2.2. Role of Autophagy in Promoting Viral Assembly and Release

Upon replication of the viral genome, it has to be packaged into a virus particle (assembly) that is released from the infected cell (exit). For many viruses, autophagy has been reported to regulate also these late steps of the viral replication cycle [18,28,78]. For instance, in the case of hepatitis B virus (HBV), efficient envelopment of viral particles was shown to depend on induction of autophagy [88] (Figure 4). Cells that are deficient in autophagy produce significantly lower amounts of nucleic acid-containing intra- and extracellular enveloped particles. Furthermore, the major HBV envelope protein (HBsAg) was shown to bind to and colocalize with LC3-I and LC3-II during HBV infection or upon ectopic expression of HBsAg, indicating that this interaction might be important for acquiring the viral envelope. Autophagosomes might serve as membrane source; alternatively, intermediate compartments might be used to transport HBV to the site of envelopment, which is thought to occur at a post-ER/ pre-Golgi site [88].

Upon infection of macrophages with HIV-1, the viral assembly protein Gag interacts with LC3-II, and this association allows the processing of the Gag subunit p24. Confocal microscopy and EM analyses revealed that gag-derived matrix (MA) protein p17 (a component of HIV-1 particles) colocalized with membrane compartments that stained positive for LC3 and that were in close proximity to the plasma membrane [28] (Figure 4). These structures are similar to those proposed previously as sites of HIV-1 assembly and budding in macrophages [28]. Furthermore, the ability of the HIV-1 Nef protein to inhibit autophagosome acidification was also shown to be required for release of infectious virions [28]. How acidification affects Gag-LC3 interaction still remains to be addressed. 

Lastly, autophagy has been shown to serve in the non-lytic release of non-enveloped picornaviruses [18,73] (Figure 4). Pharmacological inhibition of autophagy or silencing of expression of key autophagy factors results in a decrease in infectious particle production. Moreover, disrupting the microtubule network by using nocodazole treatment or challenging cells with a mutant virus resulted in increased mobility of poliovirus-induced GFP-LC3 structures, concomitant with an increase of extracellular infectious particles [78]. Although the mechanism of this non-lytic virus release is not known, recent studies have confirmed the role of autophagy in the unconventional secretion of diverse substrates [89].

#### 3.2.3. Role of Autophagy in Suppression of Anti-Viral Innate Immunity

Several recent studies have highlighted how viruses manipulate autophagy to dampen key molecules of the innate as well as the inflammatory (NF-kB-dependent) immune response. In an elegant study by Fliss and co-workers, murine cytomegalovirus (MCMV) infection-induced autophagy was shown to selectively target NEMO (NF-kB essential modulator) to degradation in lysosomes, thus inhibiting an inflammatory response [90] (Figure 4). The authors show that inhibition occurs via an interaction of the viral protein M45 with NEMO that is required to selectively relocalize NEMO to GFP-LC3-positive autolysosomal structures [90].

A recent report by Gre´goire *et al.* showed that the IRGM protein (immunity-associated GTPase family M) is a common target of several RNA viruses to subvert the autophagy network [91]. By using a yeast two-hybrid screening approach and bioinformatic analyses. the authors determined the ability of 83 proteins from different RNA virus families to interact with 44 human autophagy-associated proteins. It turned out that the autophagy network is highly targeted by RNA viruses. Importantly, IRGM, the most frequently targeted protein, interacted with the autophagy proteins Atg5, Atg10, MAP1CL3C and SH3GLB1 (SH3-domain GRB2-like endophilin B1). Silencing of IRGM expression impaired both Measles virus (MeV), HCV and HIV-1 induced autophagy and virion production. Furthermore, IRGM interacted with MeV capsid, HCV NS3 and HIV Nef. These interactions were sufficient to induce autophagy via an IRGM-dependent pathway. This work revealed a primary role of IRGM in virus-induced autophagy and identified a large range of RNA viruses that use common strategies to manipulate autophagy in order to promote viral replication [91].

As alluded to in the previous section, the direct contribution of autophagy to promoting HCV replication is discussed controversially. However, two recent studies provided insights as to how autophagy might promote HCV replication indirectly, namely by blocking the innate immune response. The study by Ke and Chen demonstrated that UPR induced by HCV activates the complete autophagy pathway, which is required for efficient viral replication [36]. Importantly, this enhancement of replication correlated with suppression of innate antiviral immunity. Interestingly, the analogous activation of UPR and inhibition of IFN-β induction was found with a PAMP derived from DENV, arguing that both viruses utilize the same strategy to maximize RNA replication and to suppress the innate antiviral defense (Figure 4). In agreement with these conclusions, Shrivastava and coworkers found that gene silencing of Beclin 1 and Atg7 in immortalized human hepatocytes not only reduced HCV RNA replication, but also resulted in the activation of IFN signaling [82]. These studies reveal a novel mechanism employed by HCV and DENV to evade an anti-viral innate immune response. 

## 4. Future Directions

Organisms have developed highly complex defense networks against invading pathogens, and in turn, pathogens either disable or manipulate host defenses to hijack their host and eventually to co-exist with it. As described in this review, autophagy fits into this theme, because on one hand, it can promote viral replication; on the other hand, it contributes to innate and adaptive immunity and thus, limits pathogen replication. Obviously as a result of co-evolution, viruses developed multiple strategies to evade autophagy or—more extremely—to reverse the antiviral effects of autophagy into proviral ones. In spite of the tremendous progress made in the last couple of years, many important aspects remain to be addressed. For instance, autophagy selectively degrades viruses, but can viruses induce autophagy to selectively degrade antiviral platforms like mitochondria or peroxisomes? Such events are plausible considering the mitochondrial or peroxisomal localization of essential innate signaling molecules, such as MAVS (mitochondrial antiviral-signaling protein) or TRIF [92,93]. Another important question relates to selective autophagy. This process requires cargo-binding adaptor proteins, which all have a molecular signature, termed the ‘LIR’ motif. It enables interaction with LC3 in order to feed bound cargo to the autophagosome. Since viruses can also induce selective autophagy, it will be important to investigate if viral proteins can directly bind LC3 and whether this interaction is LIR dependent. Studies of these and many other aspects will certainly foster our understanding to the pathogen-host interaction, but might also open new avenues for the development of novel prophylactic or therapeutic antiviral strategies.
